# Fibroblast-derived CXCL12 regulates PTEN expression and is associated with the proliferation and invasion of colon cancer cells via PI3k/Akt signaling

**DOI:** 10.1186/s12964-019-0432-5

**Published:** 2019-09-10

**Authors:** Jiachi Ma, Xiaowen Sun, Yimin Wang, Bangling Chen, Liyu Qian, Yaguo Wang

**Affiliations:** 1 Department of Oncological Surgery, The First Affiliated Hospital of Bengbu Medical College, 287 Chang Huai Road, BengBu, 233000 AnHui People’s Republic of China; 2 Department of Dermatology, The First Affiliated Hospital of Bengbu Medical College, BengBu, 233000 AnHui People’s Republic of China

**Keywords:** Colorectal cancer, CXCL12/SDF-1α, PTEN, Proliferation, Invasiveness

## Abstract

**Background:**

Stromal-derived CXCL12 play an important role which influence the proliferation and invasiveness of colon cancer in microenvironment. The present study aimed to analyze the underlying mechanism by which CXCL12 and tumour suppressor protein phosphatase and tensin homologue deleted on chromosome 10 (PTEN) influences the metastatic potential of colon cancer and internal relation of colon cancer and stromal cells.

**Methods:**

RT-PCR and western blot were detected the expression of CXCL12, CXCR4 and PTEN in colon cancer cells and stromal cells. The co-operative effects of CXCL12 and PTEN on proliferation and invasion of colon cancer cells were evaluated by real-time PCR, proliferation and invasion assays using an in vitro system consisting of co-cultured cancer cells and stromal cells. We eventually investigated activation of PI3K/Akt signaling by CXCL12 regulate PTEN and involved in the metastatic process of colon cancer. In addition, we also examine how the knockdown of PTEN influences proliferation and invasion and correlate with CXCL12/CXCR4/PI3K/Akt, determination of PTEN up-down-stream targets that preferentially contribute to tumorigenesis.

**Results:**

Blockage of PTEN phosphorylation led to a stronger enhancement of cell proliferation and invasion upon stimulation with CXCL12 via its activation of the PI3K/Akt signaling pathway. Furthermore, knockdown of PTEN by siRNA transfection was also found to enhance the activation of the PI3K/Akt pathway, thereby promoting cell invasion and proliferation. CXCL12 induced transcriptional down-regulation of activated PTEN and this signaling pathway promotes cell survival. CXCL12/CXCR4/PI3K/Akt cascade may be critical for colon cancer cells to metastasize.

**Conclusions:**

Based on our results, we suggest that the modification of CXCR4, PTEN, or PI3K function might be promising new therapeutic approaches to inhibit the aggressive spread of colon cancer.

## Background

Colorectal cancer accounts for almost one million new cancer cases and causes a half million deaths annually worldwide. It is the third most common type of newly diagnosed cancer in both males and females and ranks third as a cause of cancer-related death [1].The present treatment for colorectal cancer is surgical ablation, but many colorectal cancers are diagnosed at a late stage, when surgical intervention is no longer effective at curing the disease. At least 40% of patients with colorectal cancer develop metastases [2], and there are no highly effective approaches against disseminated colorectal cancer. Therefore, new, non-surgical therapeutic strategies are urgently needed for the treatment of advanced or metastatic colorectal cancer. There have not been highly effective approaches against metastasis of colorectal cancer so far.

Among the several genetic changes and signaling pathways known to be involved in the development and progression of cancer, one of the most common is mutation in the tumor suppressor PTEN (phosphatase and tensin homolog deleted on chromosome ten), which encodes a protein and lipid phosphatase [3]. The mutant of PTEN is unable to dephosphorylate phosphatidylinositol 3,4,5-triphosphate (PIP_3)_ which is produced by PI3K resulting in elevated intracellular PIP_3_ levels. PIP_3_, as an important messenger, transduces signals from growth factors, hormones and extracellular matrix components. One of the best-studied downstream targets of PIP_3_ is Akt, also known as protein kinase B [4]. When cells are stimulated, Akt is recruited by PIP_3_ to the plasma membrane, where Akt is phosphorylated and activated. Since PI3K/Akt signaling is involved in promoting cell survival, proliferation and migration [5, 6]. PI3K and Akt can themselves also become hyperactivated due to gene amplification or PTEN inactivation. Consequently, the downstream targets of PI3K/Akt can be abnormally activated, thereby promoting proliferation and survival of cancer cells during carcinogenesis [7]. Nuclear factor κB (NF-κB) and activator protein 1 (AP-1) are the transcription factor, and also are the targets of the Akt pathway whose activation is most strongly correlated with carcinogenesis [8]. PTEN inhibits downstream functions mediated by the PI3K pathway, such as cell growth, survival, migration, and invasiveness [9], and cell cycle progression, through the regulation of the expression of the cyclin-dependent kinase inhibitor protein p27, which is induced by PTEN in various cell types [10]. PTEN contains a sequence motif that is highly conserved in the members of the protein tyrosine phosphatase family [11]. PTEN is frequently affected in cancer, and inherited PTEN mutation causes cancer-susceptibility condition such as Cowden Syndrome. PTEN is also frequently mutated in other human cancers, including breast, lung, prostate, bladder and glioblast cancer [12]. PTEN mutations have been mapped to the conserved phospatase catalytic domain, suggesting that the phosphatase activity of PTEN is required for its tumor suppressing function.

The chemokine CXCL12 is a potent chemokines for hematopoetic cells, also known as stromal-derived factor-1 (SDF-1) [13]. CXCR4 has been shown to be a key receptor in mediating the metastasis of multiple types of tumors. Binding of CXCL12 to CXCR4 induces trimeric G protein signaling leading to activation of PI3K and JNK pathways, contributing to protease production and cellular migration and invasion. In addition, we recently found that epidermal growth factor receptor family members are activated downstream of CXCL12/CXCR4 signaling providing proliferation signals in bone tumor growth. CXCL12 and its receptors have been strongly linked to prostate cancer bone metastasis and are markers for poor prognosis [14, 15]. The other studies have shown that CXCL12/CXCR4 related axis are involved in tumor metastasis to sites which are characterized by high production of CXCL12, such as lung and bone marrow [16]. The activation of the CXCL12/CXCR4 signaling axis leads to chemotaxis, cell survival, and proliferation, however, the downstream signaling cascades are tissue-specific and not well characterized in colon cancer [17].

## Materials and methods

### Reagents and antibody

Recombinant Human CXCL12 and anti-human CXCL12 antibody were purchased by R&D system Inc. (Minneapolis, MN, USA). LY294002 (PI3K inhibitor) was ordered from Cell Signaling Technology (Beverly, MA, USA). The monoclonal antibodies (mAbs) included PTEN antibody, phospho-PTEN (ser380) antibody, Akt antibody, phospho-Akt (ser473), PI3K p85 antibody, phospho-PI3K p85 (Tyr 458) /p55 (Tyr199) antibody were provided by Cell Signaling Technology.

### Cell culture

Human colon carcinoma cell lines were purchased by American Type Culture Collection (Rockville, MD, USA). HT-29 cells were cultured in McCoy’s supplemented with 10% fetal bovine serum (FBS). CaCo-2 were maintained in minimum essential medium eagle (Sigma Chemical Co., St. Louis, MO, USA) with high glucose and 10% FBS. Colo320 was maintained in RPMI-1640 medium (Sigma Chemical Co.) supplemented with 10% FBS. Human umbilical vein endothelial cells (HUVECs) and fibroblasts were purchased by Kurabo Co. (Osaka, Japan). HUVECs were incubated in HuMedia-EB2 medium supplemented with 2% FBS, 5 ng/ml bFGF, 10 mg/ml heparin, 10 ng/ml epidermal growth factor, and 1 mg/ml hydrocortisone. Fibroblasts were cultured in FBM-2medium supplemented with 2% FBS, 1 ng/ml bFGF, and 1 mg/ml insulin. All cells were incubated at 37 °C in a humidified atmosphere of 5% CO_2_ in air.

### RT-PCR analysis

The total RNA was extracted from colon cancer cells and fibroblasts by an Isogen Kit (Nippon Gene, Tokyo, Japan), and quantities determined spectrophotometrically. The total RNA aliquots (5 μg) were pretreated by random hexamers and dNTP mix were incubated at 65 °C for 5 min, chilled on ice, and reverse transcribed into cDNA by cDNA Synthesis Mix. The 1 μl of cDNA was used for amplifcation reaction; the operations were carried out according to the supplier’s instructions. Primer sequence and PCR condition are shown in Table 1.

**Table 1:** Primer sequence and PCR condition

*Gene name*	*Primer sequences*	*Tm(°C)*	*Cycles*	*Length(bp)*	*Accession number*
PTEN	F: 5′-ACCAGGACCAGAGGAAACCT-3′ R: 5′-GCTAGCCTCTGGATTTGACG-3’	58	35	241	NM-000314
CXCL12	F: 5’-TTCCATTTGCAAGGGAAAAG-3′ R: 5′-ACACACAGCCAGTCAACGAG-3’	56	35	236	NM-000609
CXCR4	F: 5’-GAAGCTGTTGGCTGAAAAGG-3′ R: 5′-GAGTCGATGCTGATCCCAAT-3′	54	35	345	NM-003467

### Real-time quantitative RT-PCR

The PCR was conducted by LightCycler apparatus. First, the 1 μl of total RNA was added to 1 μl oligo dT primer (50 μM), and the mixture was incubated at 37 °C for 15 min at 85 °C for 5 s to reverse transcription. The PCR was carried out in a 20 μl final volume containing the following: H_2_O up to 20 μl, 10 μl TaqMan® Universal PCR Master Mix, No AmpErase® UNG (2×)^2^–ordered separately, 1 μl of 20 × TaqMan® Gene Expression Assay Mix, and 9 μl cDNA diluted in RNase-Free water. After an initial denaturation step at 94 °C for 15 s, temperature cycling was initiated. Each cycle consisted of denaturation at 95 °C for 10 s, hybridization at 60 °C for 30 s, and elongation at 72 °C for 30s. The fluorescence signal was acquired at the end of the hybridization step. The total of 45–50 cycles were performed. Melting curves were obtained for the temperature range 65 °C to 95 °C, read every 0.2 °C, hold for 5 s, then, incubate at 65 °C for 60s. Cycling conditions for GAPDH were the same as mentioned above. For each run, a standard curve was constructed from serial dilutions of cDNA from the HT-29 cell line. The level of expression of PTEN mRNA is given as relative copy numbers normalized against GAPDH mRNA and shown as mean ± standard deviation (s.d.). Relative PTEN mRNA expression was calculated using the formula (A/G÷A_0_/G_0_), where A is the relative copy numbers of PTEN mRNA; G is the relative copy number of GAPDH mRNA, A_0_and G_0_ are relative PTEN and GAPDH mRNA from the standard cDNA dilutions as a non-template control.

### Western blot analysis

The cells were lysed by lysis buffer [25 mM Tris (pH 7.8) with H_3_PO, 2 mM CDTA, 10 mM DTT, 10% glycerol, 1% Triton® X-100, 2 mM PMSF, 1 mM sodium orthovanadate, and 10 μM leupeptin]. The concentrations of protein were measured with a BCA protein assay kit (Pierce, Rockford, USA). The 30 μg of total protein per each lane were separated by 10% SDS-polyacrylamide gel electrophoresis, transferred to polyvinylidene membrane. The membrane was incubated in the blocking buffer for 60 min at room temperature. The blocking buffer was consisted of 5% non-fat dry milk dissolved into Tris buffered saline containing 0.1% Tween 20 (TBS-T). After washing the membrane with TBS-T, the membrane was immunoblotted with each primary antibody diluted into 1:1000–2000 overnight at 4 °C. Afterward, membranes were washed with TBS-T three times, and subjected to HRP-conjugated secondary antibody for 60 min at room temperature. The antibody complexes were visualized with an ECL Western blotting detection and analysis system (Amersham Biosciences, Buckinghamshire, UK). β-actin Western blots were acted as controls.

### RNA interference (siRNA)-induced gene silencing

HT-29, Colo320 and CaCo-2 cells were transfected with siRNA for PTEN or control nonspecific siRNA using Steaith TM siRNA Duplex Oligoribouncleotides (Invitrogen). The colon cancer cells were plated at 2 × 10^5^ cells per 35-mm dish in medium with 10% FBS and without antibiotic for 24 h before transfection, grown to 90% confluence the day of transfection. Diluted 200 pmol of StealthTM PTEN siRNA oligomer or PTEN siRNA control in 500 μl of Opti-MEM® I Reduced Serum Medium (Invitrogen), then and diluted 10 μl of LipofectamineTM 2000 in 500 μl Opti-MEM® I Reduced Serum Medium. These were mixed gently and incubated for 5 min. After incubation, the diluted siRNA and diluted LipofectamineTM 2000 were combined, and mixed quickly and allowed to incubate for another 20 min. Thereafter, the culture cells were directly added with the mixed solution of siRNA: Lipofectamine™ 2000 at a concentration of 100 nmol/l and mixed homogeneously, and then the mixture was placed and cultured in an incubator at 37 °C. The cells were harvested at 48 h after transfection for subsequent experiments.

### Proliferation assay

Untransfected HT-29, Colo320, CaCo-2, transfected with PTEN siRNA and Control siRNA HT-29, Colo320 and CaCo-2 colon cancer cells in the logarithmic phase were harvested, and each type of cells divided into the transfected group (PTEN siRNA): the negative control group (Control siRNA) and the untransfected group. Cells were seeded at a density of 5 × 10^3^ cells/100 μl into 96-well flat-bottomed plates and cultured overnight. The media were changed, and the cells then cultured in the medium alone (control) or in the medium containing different concentrations of CXCL12 and anti CXCL12 antibody, and after 72 h incubation, 10 μl WST-1 reagent was added to each well and cells were incubated for another 4 h at 37 °C, then the cell proliferation was measured by the WST-1 Cell Proliferation Assay. The absorbance was determined using a microplate reader (Molecular Devices, Sunnyvale, CA, USA) at a test wavelength of 450 nm and reference wavelength of 690 nm.

### Invasion assay

The invasive capability of human colon cancer cell lines was determined by Matrigel-coated invasion chambers. This system is separated by a PET membrane coated with Matrigel Matrix such that only invasive cells can migrate through the membrane to the reverse side. After rehydration for 2 h in a humidified incubator at 37 °C with 5% CO_2_, Colon cancer cells (5 × 10^4^cells/ml) were suspended in medium containing 2% FBS and seeded into the Matrigel pre-coated transwell chambers consisting of polycarbonate membranes with 8-μm pores, and fibroblasts were seeded at a density of 2 × 10^5^cells/well into the inner chambers in 24-well plates, then the transwell chambers were then placed into 24-well plates, into which we added basal medium only or basal medium containing gradient concentrations of CXCL12. After incubating for 24 h, the upper surface of the transwell chambers was wiped with a cotton swab and the invading cells were fixed and stained with Diff-Quick stain. The number of invading cells was counted in five random microscopic fields of the low filter surface under a microscope at 200 × magnification. Each condition was assessed in triplicate.

### Measurement of Caspase-3 activity

Caspase-3 activity was measured by the caspACE™ colorimetric assay system (Promega, Madison, WI, USA) according to the manufacturer’s instructions. Briefly, all four colon cancer cells (transfected with PTEN siRNA or control siRNA) were treated with or without CXCL12. LY294002, and incubated for 24 h. The cells were harvested and resuspended in the cell lysis buffer at a density of 1 × 10^6^cells/ml. After lysis, cell extracts (50 μg protein) were mixed with 32 μl of assay buffer and 2 μl of 10 mM DEVD-pNA substrate. After incubating at 37 °C for 4 h, absorbance was measured using a microplate reader at 405 nm. Absorbance of each sample was determined by subtraction of the mean absorbance of the blank from that of the sample.

### Statistical analysis

Statistical comparisons were made using the Student’s *t*-test for paired observations or one-way ANOVA with a post hoc test (Dunnett multiple comparison) for multiple group comparisons. Statistical significance was indicated by *p <* 0.05. Data are presented as mean ± s.d. Each experiment was carried out in triplicate.

## Results

### Expression of PTEN, CXCL12 and CXCR4 in colon cancer cell lines and stromal cells

**Fig. 1:**
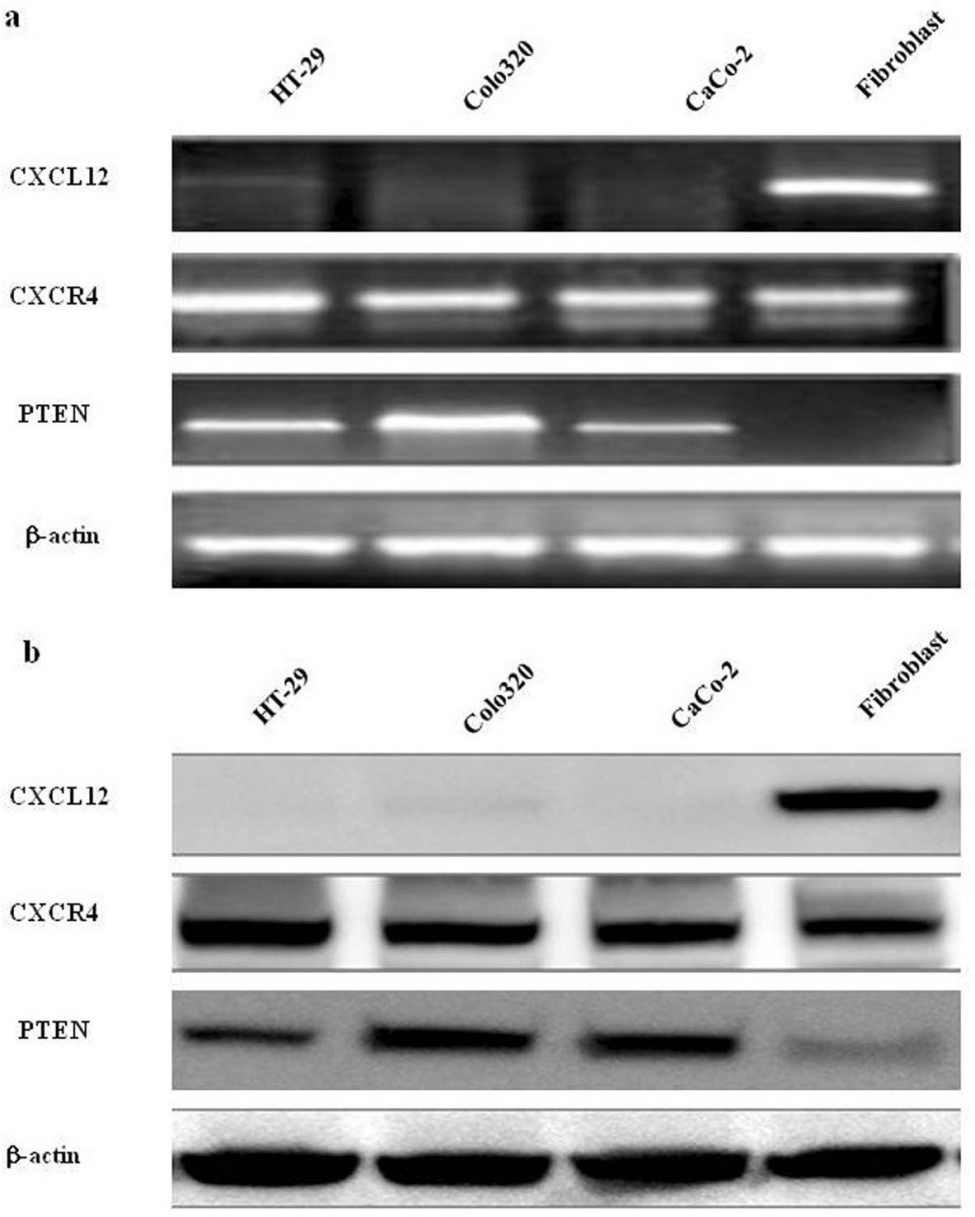
Expression levels of PTEN, CXCL12 and CXCR4 in colon cancer cell lines and stromal cells. (**a**) PTEN, CXCL12 and its receptor CXCR4 mRNA were detected by RT-PCR in colon cancer cells and fibroblasts. PCR-amplified products of reverse-transcribed mRNA (cDNA) from GenBank, using primers specific for PTEN, CXCL12 and CXCR4 PCR products, were separated through 2% agarose gels and stained with ethidium bromide. β-actin served as a loading control. (**b**) The protein expression levels of CXCL12 and CXCR4 in colon cancer cell lines and fibroblasts were determined in whole-cell lysates by Western blotting analysis. Thirty micrograms of total cell lysate was subjected to 10% SDS-PAGE and transferred to polyvinylidene difluoride membrane. The membrane was probed with antibodies to PTEN, CXCL12 and CXCR4. β-actin acted as a loading control

The expression of PTEN, CXCL12 and CXCR4 mRNA and protein were detected in colon cancer cell lines and stromal cells using RT-PCR and Western blot. RT-PCR results revealed that all colon cancer cell lines were found to express PTEN and CXCR4 mRNA. CXCL12 mRNA was only expressed in fibroblasts, but not expressed PTEN in fibroblasts (Fig. 1a). Consistent with the RT-PCR results, the immunoblotting analysis CXCL12 protein was only expressed in fibroblasts, but not in HT29, CaCo-2, Colo320. CXCR4 and PTEN were expressed in all colon cancer cell lines (Fig. 1b).

### Effect of CXCL12 and co-cultured with fibroblast on expressed level of PTEN mRNA from colon cancer cell lines

**Fig. 2:**
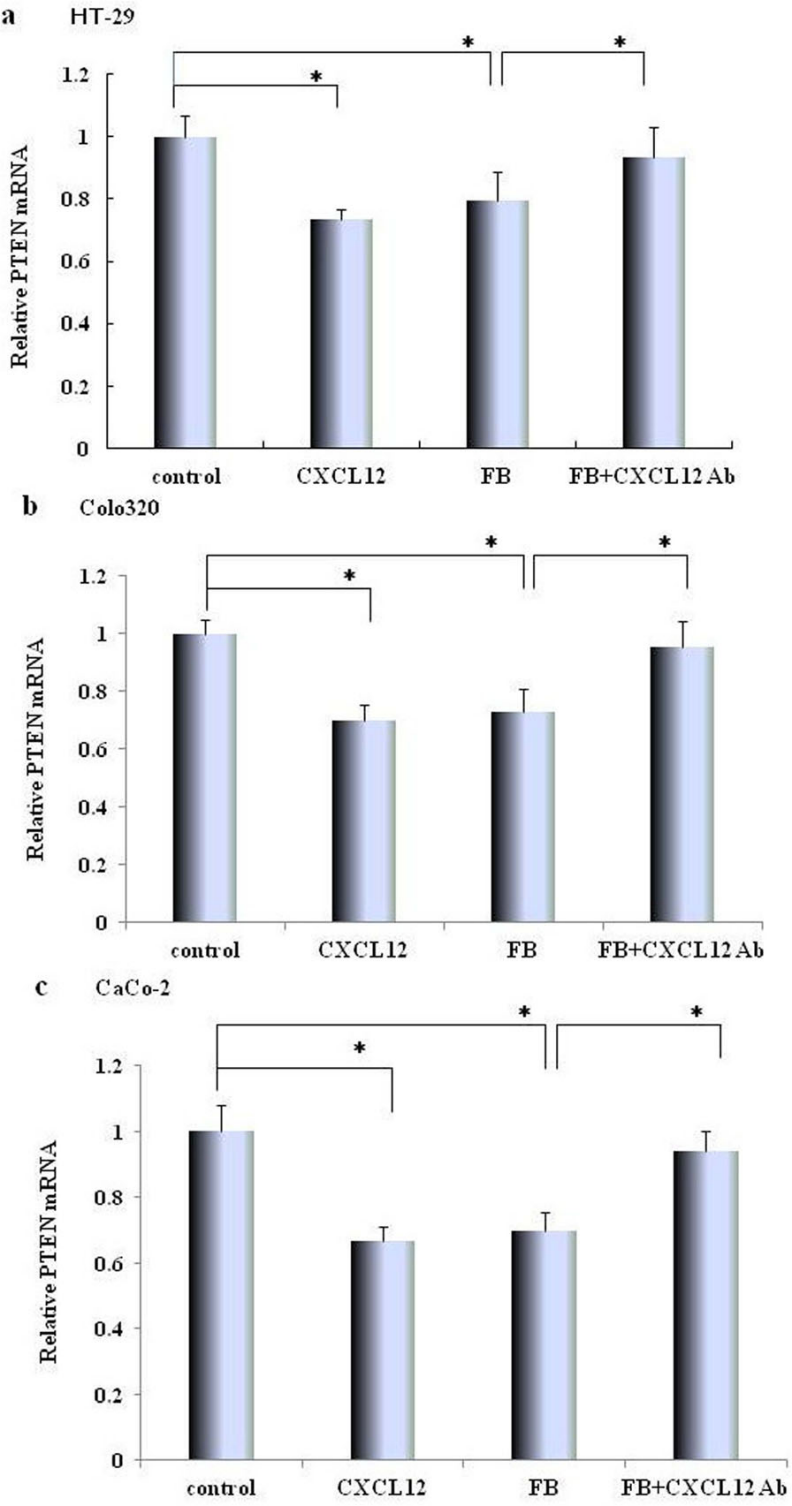
Effect of recombinant CXCL12 and co-culture with fibroblasts on PTEN Relative expression of PTEN mRNA in colon cancer cell lines. The alteration of PTEN mRNA from colon cancer cell lines[HT-29 (**a**), Colo320 (**b**), and CaCo-2 (**c**)] by recombinant CXCL12 stimulation, co-culture with fibroblasts (FB) or co-culture with fibroblasts+anti CXCL12 antibody were determined by semi-quantitative RT-PCR. The experimental detail is described in the “Materials and Methods” section. Control: colon cancer cells only; FB:co-culture with fibroblasts; CXCL12: treated with recombinant CXCL12; FB + Ab: colon cancer cells co-cultured with fibroblasts and pre-treated with anti-CXCL12 Ab. The values are expressed as mean ± SD. Multiple comparisons were performed by one-way ANOVA followed by Dunnett test. Bars indicate SD

The expressed level of PTEN mRNA was measured by quantitative real time PCR assay in.

colon cancer cell lines. The addition of recombinant CXCL12 significantly decreased expressed level of PTEN mRNA in HT-29, compared with control (0.73 ± 0.036 vs 1.0 ± 0.07, *P < 0.01,* Fig. 2a), Colo320 (0.69 ± 0.05 vs 1.0 ± 0.05, *P < 0.01,* Fig. 2b), CaCo-2 (0.66 ± 0.03 vs 1.0 ± 0.08, *P < 0.01*, Fig. 2c); The expressed level of PTEN mRNA co-cultured with fibroblasts in HT-29, (0.797 ± 0.09vs1.0 ± 0.07, *P < 0.01,* compared with control, Fig. 2a), Colo320 (0.727 ± 0.08 vs1.0 ± 0.05, *P < 0.01,* compared with control, Fig. 2b), and CaCo-2 (0.697 ± 0.06 vs 1.0 ± 0.09, *P < 0.01*, compared with control, Fig. 2c); Furthermore, the decreased PTEN mRNA of coloncancer cells were significantly promoted by co-culturing with fibroblasts in the presence of CXCL12 Ab (*P < 0.01,* compared with co-culturing with fibroblasts).

**Fig. 3:**
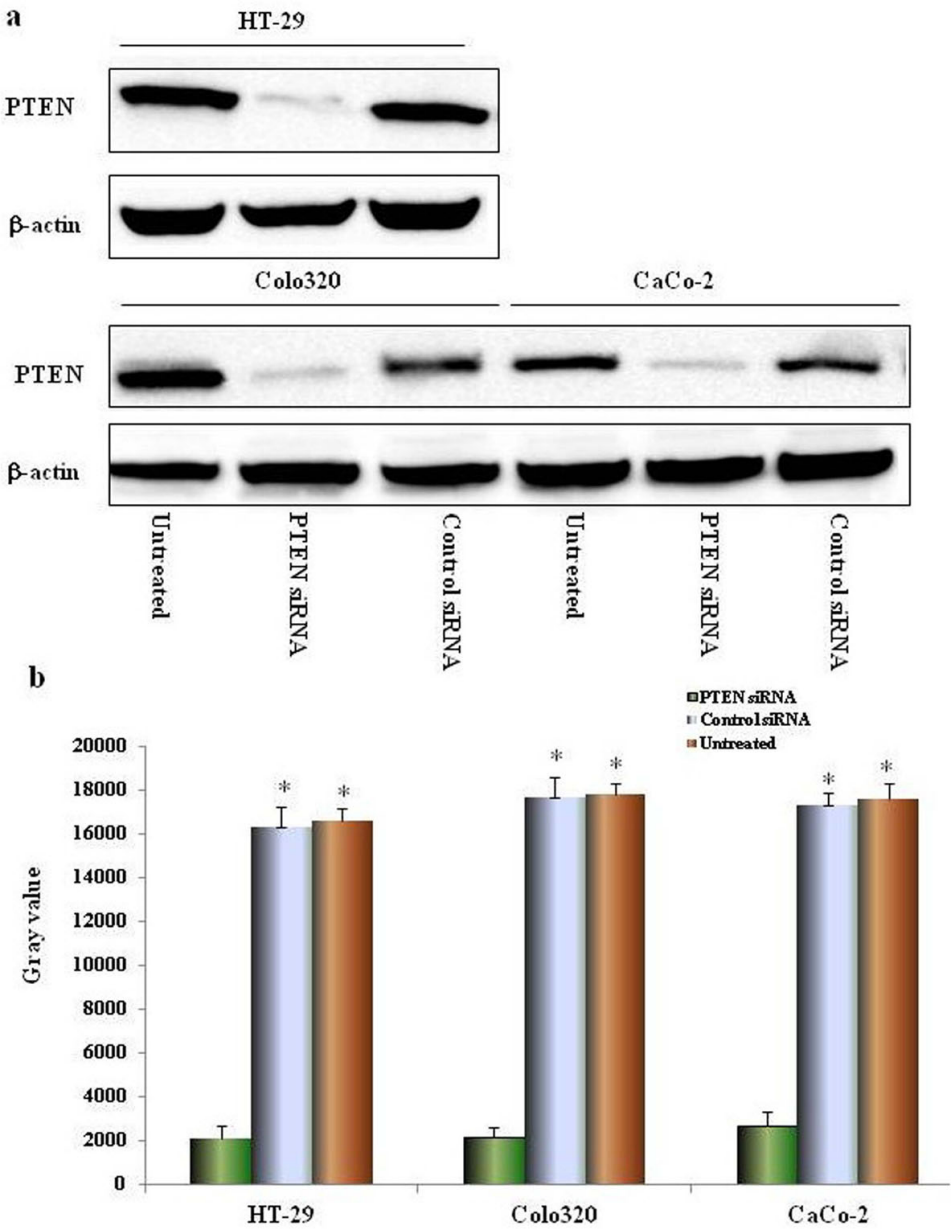
siRNA blockage of PTEN expression. The expression of CXCL12 protein in colon cancer cell line after silencing of CXCL12 gene. Knockdown of CXCL12 by CXCL12 siRNA was confrmed by immunoblotting in all three colon cancer cell lines (**a**) siRNA duplex oligoribonucleotides were transfected into cells for 48 h; the total proteins were extracted and then western blot. The grayscale values of the strips were measured by Image J software (**b**) Multiple comparisons were performed by one-way ANOVA followed by SNK test. Values are expressed as mean ± SD. Bars indicated SD. * *p* < 0.01 compared with control. Re-probing with an anti-β-actin antibody served as a control

### PTEN siRNA interference strongly downregulates expression of PTEN protein

The three human colon cancer cells were transfected with siRNA that specifically targets PTEN, the expressions of PTEN proteins was detected by western blot. The experimental results showed that: after PTEN gene silencing, compared with the untransfected and control siRNA groups and positive control β-actin (Fig. 3a), the expressions of PTEN proteins in four colon cancer cells were significantly inhibited (*P < 0.01*, respectively, compared with the untransfected and control siRNA groups), and the experiment showed that PTEN siRNA primer design and cell transfection were successful (Fig. 3b).

### Effect of CXCL12 and PTEN siRNA on the proliferation of human colon cancer cells

**Fig. 4:**
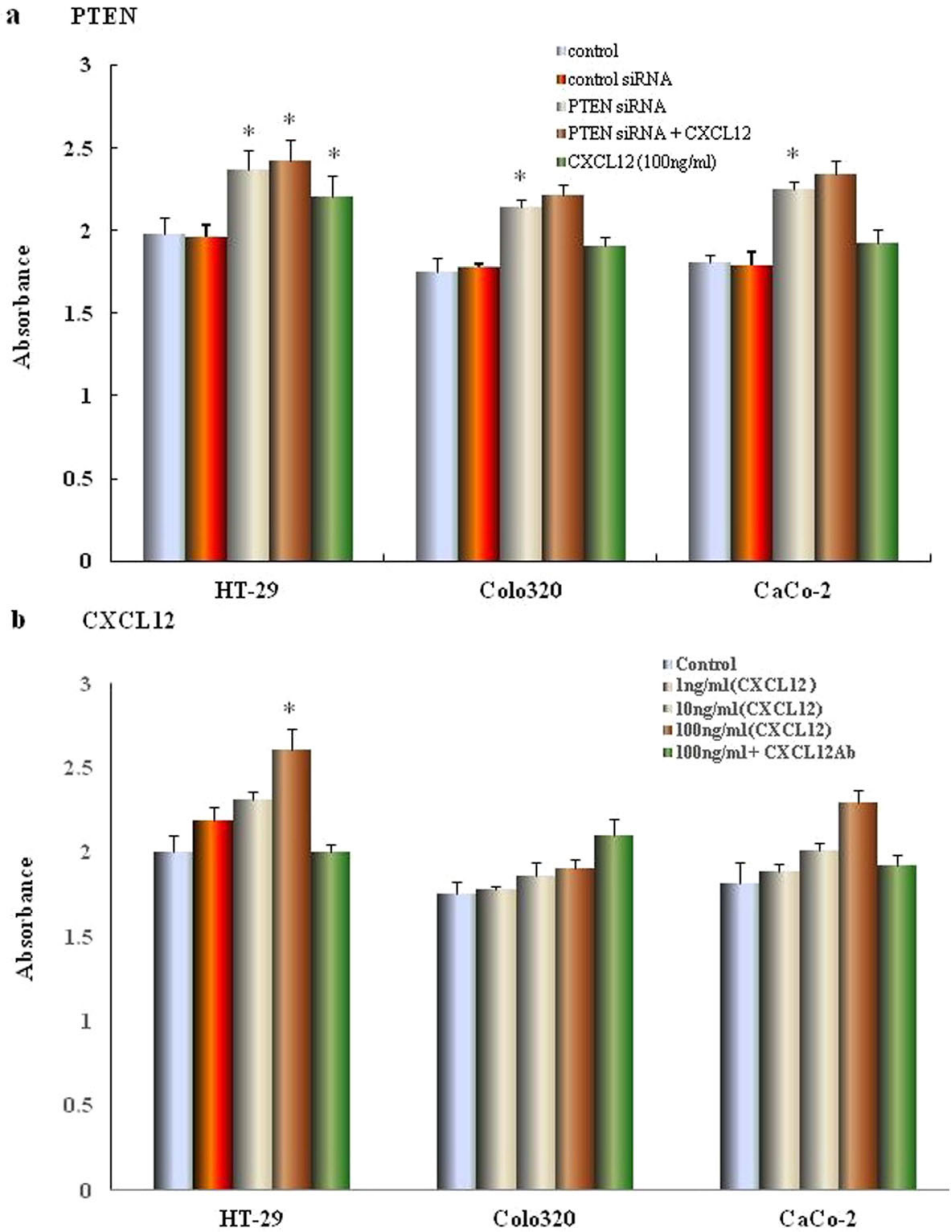
The effect of CXCL12 and PTEN gene silencing on the proliferation of colon cancer cells. (**a**) The effect of CXCL12 gene silencing on the proliferation of colon cancer cells. HT-29, CaCo-2 and Colo320 cells transfected with PTEN or control siRNA duplex oligoribonucleotide were cultured for 48 h, then cultured in the presence or absence of CXCL12 for 72 h. Cell proliferation was determined by the Premix WST-1 Cell Assay System and absorbance was read at 450 nm. The reference wavelength is 690 nm. (**b**) The effect of different concentration of recombinant CXCL12 on proliferation of colon cancer cells. HT-29, Colo320 and CaCo-2 cells were cultured in medium containing different concentrations of CXCL12. After 72 h of incubation,the proliferation of colon cancer cells were assessed using premixed WST-1 cell proliferation assay (column mean absorbance reading; Bars = SD). Multiple comparisons were performed by one-way ANOVA followed by the Dunnett test. Bars indicate SD. * *p* < 0.01, compared with control (0 ng/ml)

We next investigated colon cancer cell proliferation with and without treatment by PTEN siRNA. We also examined the proliferative effects of CXCL12 over a range of concentrations. The proliferation assay results showed that CXCL12 enhanced proliferation of the three colon cancer cell lines in a dose-dependent manner (*p < 0.01, **p < 0.05 compared with control, Fig. 4a); The addition of LY294002, an inhibitor of PI3K, inhibited the proliferation of cancer cells (*p < 0.01, **p < 0.05 compared with control, Fig. 4b). All cells transfected with PTEN siRNA, the proliferative capability was enhanced more than siRNA control cells (*p < 0.01). The capability of proliferation was also promoted by 100 ng/ml of CXCL12 in cells trefected with PTEN siRNA (*p < 0.01, compared with control siRNA, Fig. 4b).

### The roles of CXCL12 and PTEN in the invasive behavior of colon cancer cells

After pretreatment with PTEN siRNA or control siRNA, colon cancer cells were cultured with or without CXCl12 and LY294002 for 24 h. At that point, the invasive capability was assessed. CXCL12 was found to enhance the invasion of colon cancer cells in a concentration-dependent manner. The 100 ng/ml of CXCL12 was the most effective (*p < 0.01, **p < 0.05) (Tables 1). Invasive capability was higher in PTEN siRNA transfected cells than untreated cells or control siRNA-treated cells (*p < 0.01,**p < 0.05) (Table 2). On the other hand, the invasive ability was blocked by LY294002 (*p < 0.01). There was a statistical difference between PTEN siRNA and PTEN siRNA + 100 ng/ml of CXCL12 (*p < 0.01, Table 3).

**Table 2:** CXCL12 effect on invasion by colon cancer cells

Cell line	Relative number of invading cells (%)				
	Untreated	with FB	CXCL12 (ng/ml)		
			1	10	100
HT-29	100 ± 9.2	136.2 ± 10.3*	112.3 ± 8.60	132.7 ± 11.3*	162.0 ± 13.4*
CaCo-2	100 ± 11.5	139.3 ± 11.7*	109.3 ± 11.4	136.9 ± 10.5*	152.0 ± 13.9*
Colo320	100 ± 11.1	143.5 ± 12.9*	107.5 ± 10.0	127.5 ± 12.9*	148.4 ± 17.5*

NOTE: Colon cancer cells were treated with different concentrations of CXCL12 and incubated for 24 h. Cell invasion was then measured by the Matrigel assay. Statistical significance was tested by one-way ANOVA followed by the Dunnett test. Statistical significance was inferred if *p* < 0.05. All data are expressed as mean ± s.d. *p < 0.01 when compared with untreated cells

**Table 3:** The Effect of PTEN siRNA and CXCL12 on invasiveness of colon cancer cells

Cell line	Relative number of invading cells (%)					
	PTEN siRNA	Control siRNA	LY294002	PTEN siRNA + CXCL12 (ng/ml)		
				1	10	100
HT-29	147.3 ± 16.7*	100 ± 11.3	70.6 ± 13.2*	144.7 ± 17.3	152.5 ± 17.3	186.0 ± 21.3*
WiDr	128.5 ± 9.63*	100 ± 12.0	68.1 ± 10.7*	127.2 ± 10.2	136.0 ± 16.3	176.3 ± 22.2*
CaCo-2	134.0 ± 18.0*	100 ± 16.4	61.3 ± 10.1*	136.2 ± 15.2	145.2 ± 12.8	153.7 ± 30.6*
Colo320	111.9 ± 9.14	100 ± 10.4	59.1 ± 10.1*	113.1 ± 20.3	122.6 ± 21.5	156.4 ± 15.0*

NOTE: After transfection with PTEN siRNA or control siRNA, colon cancer cells were treated with different concentrations of CXCL12 and incubated for 24 h, followed by measurement of cell invasion by a Matrigel assay. Statistical significance was tested by one-way ANOVA followed by the Dunnett test. Statistical significance was inferred if *p* < 0.05. All data are expressed as mean ± s.d. *p < 0.01 when compared with the control siRNA group

### Effect of CXCL12 antibody on colon cancer cell apoptosis

**Fig. 5:**
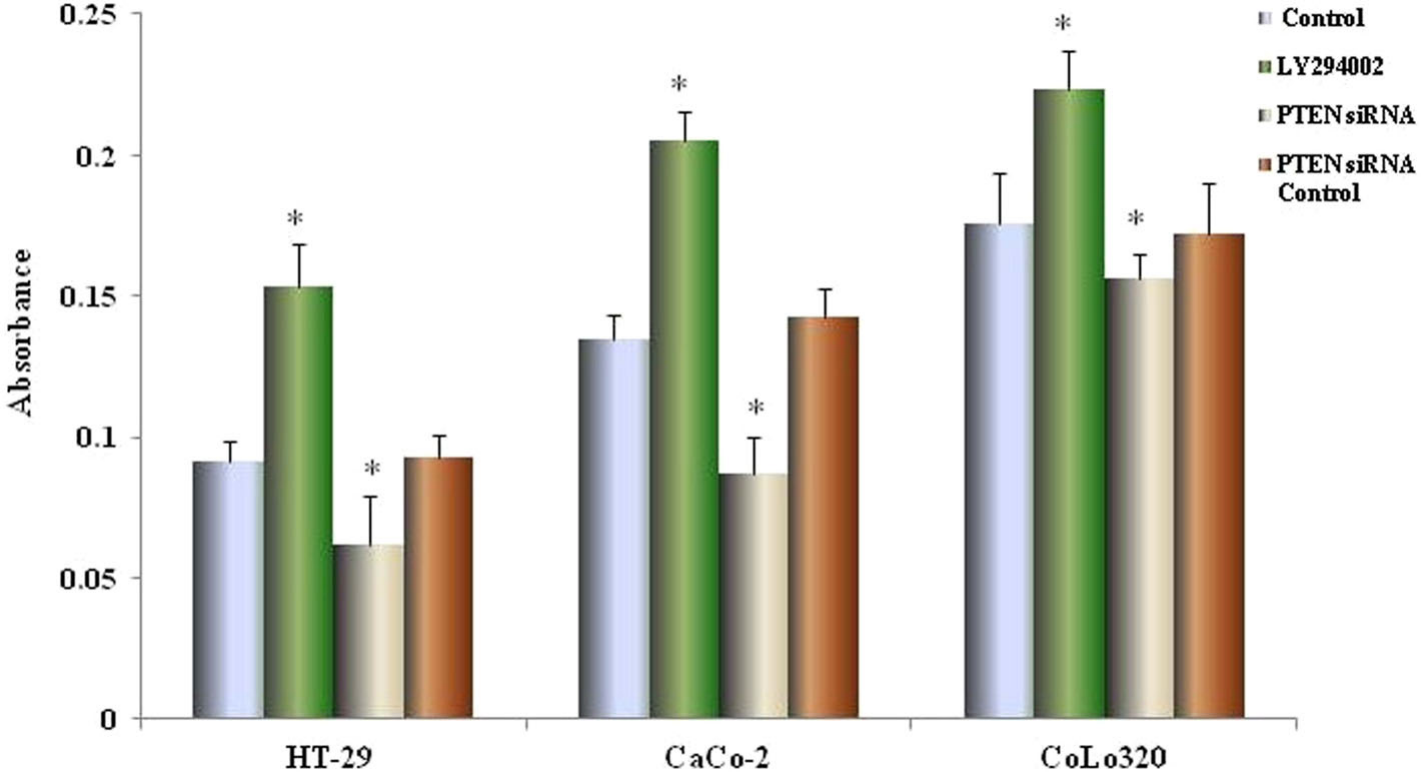
Effects of LY294002 and PTEN siRNA on apoptosis in colon cancer cells. Colon cancer cells were treated with PI3K inhibitor (LY294002), PTEN siRNA, or control siRNA and incubated for 24 h, followed by detection of apoptosis using the CaspACE™ Assay System, with absorbance being measured in the wells at 405 nm. Statistical significance was tested by one-way ANOVA followed by Dunnett test. The *p*-values indicate statistical significance between control and experimental data sets. Bars indicate the s.d. *p < 0.01 compared with control

Colon cancer cells were treated with LY294002, PTEN siRNA and control siRNA, and then incubated for 24 h, the apoptosis was measured by the CaspACE™ assay. LY294002 significantly increased apoptosis of colon cancer cells (*p < 0.01 compared with control). In contrast, the apoptosis of colon cancer cells was inhibited by PTEN siRNA transfection (*p < 0.01 compared with siRNA control) (Fig. 5).

### Activation of the PI3K and Akt signaling pathway after CXCL12 stimulation in human colon cancer cells

**Fig. 6:**
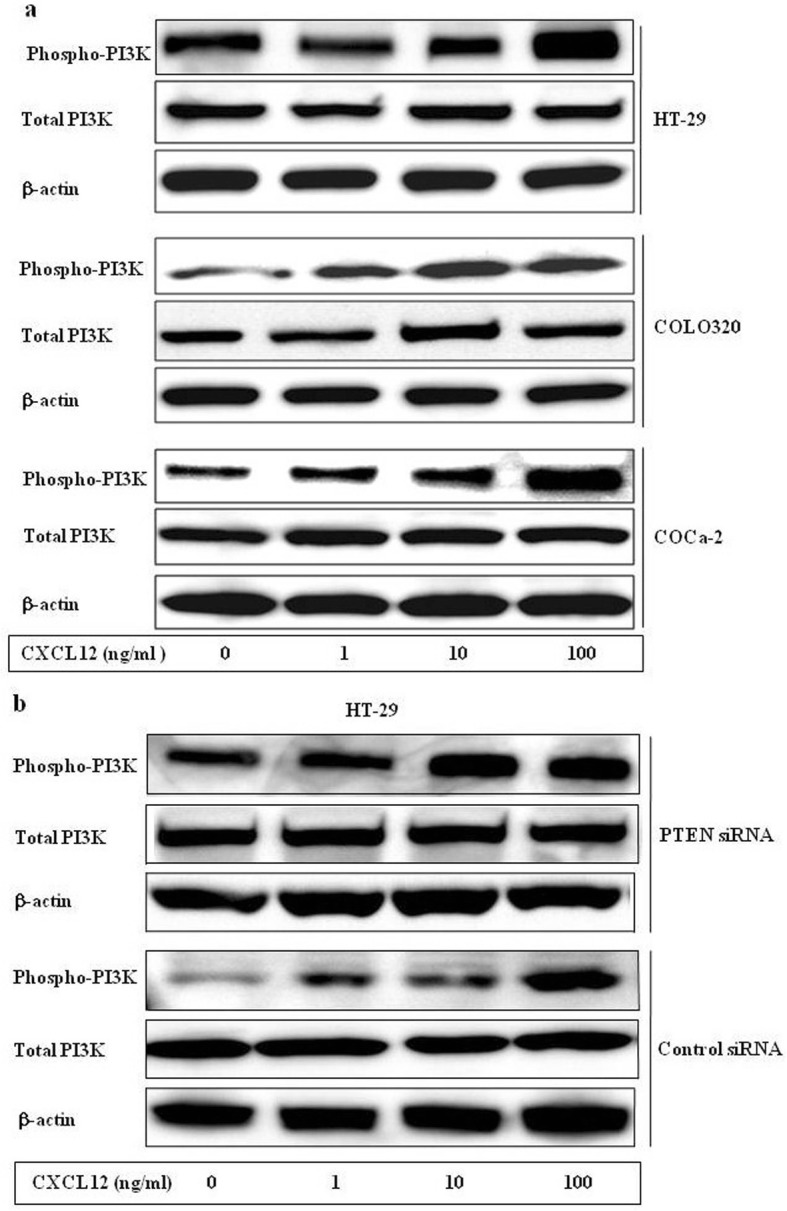
The different concentration of CXCL12 activates phosphorylation of PI3K in HT-29, Colo320, CaCo-2 and PTEN siRNA transfected HT-29 cells. (**a**) HT-29, Colo320, CaCo-2 cells were treated with 1 ng/ml, 10 ng/ml and100ng/ml of CXCL12, and incubated for 15 min. The cells were collected from each time point, lysed by lysis buffer, and immunoblotted with a phospho-PI3K antibody as described in Materials and Methods. Detection of total PI3K levels served as a loading control. (**b**) The different concentration of CXCL12 affects phosphorylation of PI3K and total PI3K in the PTEN siRNA transfected and control siRNA HT-29 cells. β-actin served as a loading control

We used the colon cancer cell lines to examine the activation of the PI3K/Akt signaling pathway, a downstream target of CXCl12. CXCl12 treatment increased PI3K phosphorylation in a dose-dependent manner in HT-29, WiDr, CaCo-21 and Colo320 cells (Fig. 6a). We also examined the response to CXCl12 in HT-29 cells which had been transfected with PTEN siRNA. The data indicate that phosphorylation of PI3K was enhanced in PTEN siRNA transfected cells more than in untransfected cells (Fig. 6b). This result demonstrates that PTEN protein could reduce PI3K phosphorylation in colon cancer cells.

**Fig. 7:**
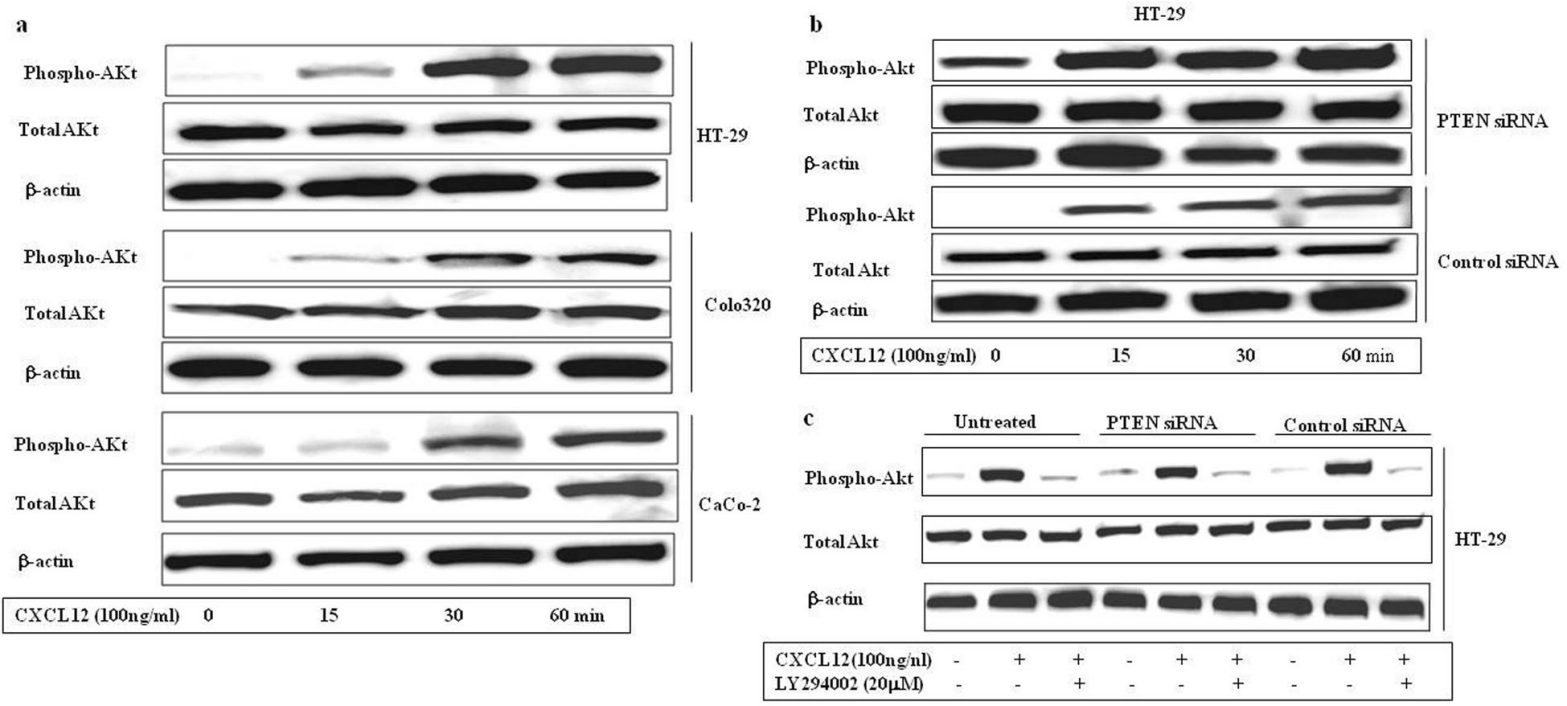
CXCL12-induced phosphorylation of Akt in human colon cancer cell lines. (**a**) The different concentration of CXCL12 activates phosphorylation of PI3K in HT-29, Colo32 0and CaC-2 cells. The cells were treated with different concentrations of CXCL12 (100 ng/ml) and cultured for for 0, 15, 30, or 60 min. The cells were gathered and lysed by lysis buffer. The 30 micrograms of lysed protein were used to do immunoblotting with a phospho-Akt antibody. Detection of total Akt levels served as a loading control. (**b**) Expression of phospho-Akt and total Akt in both PTEN siRNA treated and control siRNA treated HT-29 cells. (**c**) LY294002 inhibited Akt expression in untreated, PTEN siRNA transfected and control siRNA HT-29 cells

The Akt kinase activity of colon cancer cells was remarkably enhanced by CXCL12 stimulation in a time-dependent manner (Fig. 7a). Stronger activation of Akt kinase activity was observed in HT-29 cells which had been transfected with PTEN siRNA (Fig. 7b). In contrast, LY294002 suppressed Akt kinase activation (Fig. 7c). Not only does CXCL12 stimulate Akt kinase activity, but that stimulation is enhanced by treatment with PTEN siRNA.

## Discussion

Epidemiologic studies have shown that high levels of CXCL12 are associated with increased risk for several common cancers, including those of breast, gastric cancer, hepatocellular carcinoma and colorectal cancers [18–21]. Functionally, CXCL12 not only stimulates cell proliferation but also promotes cell migration. In this study, we demonstrated that stromal cell-derived CXCL12 induced the suppression of expressed PTEN and enhanced both proliferation and invasion through the activated CXCL12/CXCR4/PI3K/Akt signaling pathway in colon cancer cells. Similarly, knockdown of PTEN expression with siRNA interference also led to the enhancement of PI3K/Akt signaling strongly implicating PTEN as a regulator of Akt signaling in colon cancer.

In our previous studies, we classified that IGF-1 induced the dephosphorylation of PTEN and up-regulated cellular invasiveness and proliferation through PI3K–PTEN–Akt–NFkappaB signaling pathway. We also found a negative correlation between PTEN expression and liver metastasis in pancreatic cancer cells. Knockdown of PTEN enhanced the invasiveness and proliferation of pancreatic cancer cells [22, 23]. Several studies have demonstrated that the overexpression of PTEN inhibits cell growth in a variety of cancer cell lines [24]. Furthermore, it is well known that PTEN is suppressed in a variety of cancers and that PTEN protein plays an important role in the carcinogenesis of multiple human cancer cells, including colorectal cancer [25]. PTEN expression is decreased in colorectal cancers compared with its expression in polyps and normal mucosa. This is consistent with evidence suggesting that PTEN expression is decreased in approximately 40% colorectal cancers, often in association with a PTEN mutation or deletion [26]. In addition to colorectal cancer, the loss or reduced expression of PTEN has been found to occur in other cancers, most notably breast, prostate and gastric carcinomas [27, 28]. Furthermore, the expression of PTEN protein was found to be decreased in the distal colon and rectum in animal studies overexpression of PTEN in colorectal cancer cells has been found to result in cell cycle arrest and enhanced cell death through the inhibition of PI3K [29]. It is interesting to speculate whether decreased PTEN expression may contribute to propensity for cancer in the more distal colon and rectum. In our results, the expression levels of PTEN mRNA is significantly reduced in highly liver metastatic colon cancer cell line HT-29 than in low liver metastatic colon cancer cell lines CaCo-2 and Colo320.

Our data highlights a co-ordinated response of tumor and stromal cells in the microenvironment. As key signaling molecules in tumor microenvironment, the fibroblast-derived CXCL12 has been shown to play an important role in the development of colon cancer. CXCL12 and its specifc receptors CXCR4 have been shown to be associated with the growth and metastasis of a variety of malignant tumors [30–32]. The other studies have shown that the expressions of CXCL12 and CXCR4 in colon cancer patients are associated with liver metastasis, recurrence rate and survival rate in colorectal cancer patients [33]. While the inhibition of CXCL12 and CXCR4 can signifcantly reduce tumor cell proliferation and metastasis [34], indicating that CXCL12 is closely related to development, outcome and prognosis of colon cancers. The RNA interference used to systematically examine the role of PTEN in proliferation, invasion and apoptosis in colon cancer cell lines. We found that loss of PTEN can enhance proliferation and invasion and thereby the invasive potential is promoted. This suggests that the lower expression of PTEN in high liver metastatic cell lines may be a key reason those lines are more metastatic. Furthermore, the fibroblasts-derived of CXCL12 blockage of PTEN expression in microenvironment; in other words, there is a correlation between the reduction in active PTEN and increase in downstream signalling and behaviour. PTEN suppression results in the activation of PI3K/Akt pathway and its downstream target. PI3K inhibitor activated PTEN phosphorylation, in turn blocking PI3K and downstream targets, and consequently inhibited the proliferation and invasion in colon cancer cells. To better investigate the mechanism by which PTEN affects metastatic potential in colon cancer, we evaluated the knockdown of PTEN by RNA interference-induced PTEN gene silencing, and found that blockage of PTEN expression not only enhanced the activity of PI3K and its downstream targets Akt, but also promoted proliferation and invasion in colon cancer cell lines. Akt is a downstream target of PI3K and the PI3K/Akt pathway has recently been recognized as one of the most important signals ensuring protection against apoptosis [33]. Therefore, CXCL12/CXCR4/PI3K/Akt signaling pathway has become a hot topic in tumor research and also provides a specifc target spot for cancer treatment.

In summary, we have demonstrated that stromal cell-derived CXCL12 to activate PI3K/Akt pathway by down-regulating PTEN leads to enhanced proliferation and invasion in colon cancer cells. Inhibition of microenvironmental CXCL12/CXCR4 signaling may be one approach by which to enhance PTEN phosphorylation, which inhibits colon cancer cell growth. The results reported herein indicate that inhibition of PI3K phosphorylation may be a major mechanism by which CXCL12 antibody and LY294002 inhibits cancer cell proliferation and invasion and induces apoptosis. Based on our findings, we speculate that PTEN suppresses cell growth, at least in part, through disturbing the function of CXCL12 in colon cancers.

## Conclusion

In this study, we provide evidence that CXCL12/CXCR4/PI3K/Akt cascade may be critical for colon cancer cells to metastasize. Based on our results, we suggest that the modification of CXCR4, PTEN, or PI3K function might be promising new therapeutic approaches to inhibit the aggressive spread of colon cancer.

## Abbreviations

Akt: Protein kinase B
AP-1: Activator protein 1
CXCL12: Stromal-derived factor-1 (SDF-1)
CXCR4: Chemokine (C-X-C motif) receptor 4
FB: Fibroblast
IGF-1: Insulin-like growth factors − 1
NF-κB: Nuclear factor κB
PI3K: Phosphatidylinositol 3-kinase
PIP_3_: Dephosphorylate phosphatidylinositol 3,4,5-triphosphate
PTEN: Phosphatase and tensin homolog deleted on chromosome ten)
